# Growth differentiation factor 15 contributes to cancer-associated fibroblasts-mediated chemo-protection of AML cells

**DOI:** 10.1186/s13046-016-0405-0

**Published:** 2016-09-19

**Authors:** Yuanmei Zhai, Jing Zhang, Hui Wang, Wei Lu, Sihong Liu, Yehua Yu, Wei Weng, Zhiyong Ding, Qi Zhu, Jun Shi

**Affiliations:** 1 Department of Hematology, Shanghai Jiao Tong University Affiliated Sixth People’s Hospital, Shanghai, 200233 China; 2 Department of Hematology, Tongren Hospital, Shanghai Jiao Tong University School of Medicine, Shanghai, 200336 China; 3 Department of Hematology, Shanghai Jiao Tong University Affiliated Sixth People’s Hospital South campus, Shanghai, 201400 China; 4 Department of Hematology, Shanghai Jiao Tong University School of Medicine Affiliated Ninth People’s Hospital, Shanghai, 200011 China

**Keywords:** Bone marrow microenvironment, CAFs, Acute myeloid leukemia, GDF15

## Abstract

**Background:**

Chemo-resistance is still a major obstacle in efforts to overcome acute myeloid leukemia (AML). An emerging concept has proposed that interactions between the bone marrow (BM) microenvironment and leukemia cells reduce the sensitivity of the leukemia cells to chemotherapy. As an important element of the tumor microenvironment, the cancer-associated fibroblasts (CAFs) are considered to be activated modulators in the chemo-resistance of many solid tumors. But their contribution to AML has yet to be fully understood. Here we report a critical role for CAFs which were thought to be a survival and chemo-protective factor for leukemia cells.

**Methods:**

A retrospective study on the BM biopsies from 63 primary AML patients and 59 normal controls was applied to quantitative analysis the fiber stroma in the BM sections. Then immunohistochemistry on the BM biopsies were used to detect the makers of the CAFs. Their effects on drug resistance of leukemia cells were further to be assessed by co-cultured experiments in vitro. Moreover, the possible mechanisms involved in CAF-mediated chemo-protection of AML cells was investigated by antibody neutralization and siRNA knockdown experiments, with particular emphasis on the role of GDF15.

**Results:**

In our study, excessive reticular fibers in the BM led to higher frequency of relapse and mortality in primary AML patients, bringing the inspiration for us to investigate the functional roles of the fiber-devied cells. We declared that the CAF cells which expressed higher levels of FSP1, α-SMA or FAP protein were widely distributed in the marrow of AML. Then in vitro co-cultured tests showed that these CAFs could protect leukemia cell lines (THP-1/K562) from chemotherapy. Interestingly, this effect could be decreased by either treatment with a neutralizing anti-GDF15 antibody or knockdown GDF15 (with siGDF15) in CAFs. Furthermore, we also confirmed that the GDF15^+^ cells mainly co-localized with FAP, which was identified as the typical phenotype of CAFs in the BM stroma.

**Conclusions:**

We firstly demonstrate that the functional CAFs are widespread within the BM of AML patients and should be a critical chemo-protective element for AML cells by producing amount of GDF15.

**Electronic supplementary material:**

The online version of this article (doi:10.1186/s13046-016-0405-0) contains supplementary material, which is available to authorized users.

## Background

Chemo-resistance remains a significant challenge in acute myeloid leukemia (AML) and is one of the critical prognostic elements. Evidence has suggested that the interaction between leukemia cells and the BM stroma could affect the chemo-sensitivity of the tumor cells [1]. As an important element of the tumor microenvironment, fibroblasts with an activated phenotype, referred to as ‘activated myofibroblasts’ or ‘cancer-associated fibroblasts’ (CAFs), have been reported to play a critical role in the chemo-resistance of solid tumors [2]. However, CAFs still appear not to be a concern in leukemia because BM biopsies are not routinely performed in AML patients. Therefore, whether CAFs exist in the BM microenvironment and whether they are involved in resistance to chemotherapy still remain unclear.

CAFs display phenotypes that are similar to those of myofibroblasts derived from quiescent fibroblasts, which are activated when they interact with carcinoma cells during tumorigenesis [3]. These powerful cells are immunologically defined primarily by the expression of α-smooth-muscle actin (α-SMA), fibroblast-activated protein (FAP), and fibroblast-specific protein 1 (FSP1) [4–6]. Datas have revealed that CAF cells are involved in the migration, growth and chemo-resistance of tumor cells in several solid tumors, including human prostate, pancreatic, colorectal carcinoma, and in hematologic malignancies, such as multiple myeloma, and the function mainly by secreting a variety of cytokines [7–9]. In the BM from mouse models of acute lymphoblastic leukemia (ALL), we have previously determined a protective niche, which is dynamically transient between Nestin^+^ and α-SMA^+^ cells,causing the production of reticular fibers in response to chemotherapy. CAF-like cells and fibers may provide chemoresistance for leukemia cells in vivo. Meanwhile, leukemia cells contribute to the protective niche formation by secreting chemotactic factors and growth factors, in particular, growth differentiation factor 15 (GDF15) [10]. GDF15, a divergent member of the TGF-β superfamily, is highly expressed only in the placenta and in macrophage cells under physiological conditions [11]. However, it is clinically associated with disease progression in numerous tumors [12–14]. Recently, it has been demonstrated that CAFs could be a novel source of GDF15 in human prostate cancer and multiple myeloma [7, 15]. We propose that CAFs within the BM of AML could also counter the chemo-sensitivity of leukemia cells by producing GDF15.

In the current study, excessive reticular fibers in the BM led to a poor prognosis in primary AML patients, making it important to investigate the functional roles of the fibroblast cells. After demonstrating the presence of CAFs in the marrow microenvironment of primary AML and their ability to protect leukemia cells from chemotherapy agents in vitro, we assessed whether GDF15 contributed to the CAF-mediated chemo-protection of AML cells either by using a neutralizing anti-GDF15 antibody or by knocking down GDF15 (siGDF15) in the CAFs as well as by investigating the distribution of GDF15 in the cytoplasm of CAFs within BM sections from AML patients. These findings were used to investigate the functional roles of CAF-derived GDF15 in chemo-resistance of leukemia and whether CAF cells, by producing GDF15, could be a survival and chemo-protective element for leukemia cells.

## Methods

### Primary AML samples and normal controls

Primary AML patients except acute promyelocytic leukemia (*n* = 63) were prospectively enrolled in our study after providing written informed consent. Cases of newly diagnosed lymphoma patients without bone marrow involvement were selected as the normal control group (*n* = 59). There were no statistical differences in age and sex between these two groups (data were not shown). The studied patients with AML received the induction/reinduction chemotherapy protocols and all the BM trephine biopsy specimens and BM aspirates were obtained from Shanghai Jiaotong University-affiliated hospitals (Shanghai, China). The patients were diagnosed according to the FAB classification. Complete remission (CR) and relapse were diagnosed according to Cheson et al [16]. And the refractory AML cases were defined in accordance with Schmid et al [17]. The AML patient characteristics are presented in Table 1. The pathologists who examined the BM samples were not participant in this study and were innocent about the group situation.

**Table 1:** Patient demographics and clinical characteristics

Characteristic	Refractory group (*n* = 25)	CR group (*n* = 38)	*P* value
Age (years)			0.261
Median age	53	60	
Range	23-82	18-80	
Sex (Male/Female)	18/7	27/11	0.935
FAB subtype	-	-	0.709
M0	1	1	
M1	1	6	
M2	8	10	
M4	8	15	
M5	5	4	
M6	1	1	
M7	1	1	

*CR* complete remission, *FAB* French-American-British Classification

### Cell culture and regents

Mesenchymal stem cells (MSCs) were obtained from the BM of primary AML patients after informed consent. The marrow was diluted twice with phosphate buffered saline (PBS), and then isolated by Ficoll-Hypaque (Axis-Shield Diagnostics, Dundee, Scotland, UK) density-gradient centrifugation. Monocytes were collected by adherence to a plastic flask and incubated for 48h in MesenCult® medium (STEMCELL Technologies, Vancouver, BC, Canada). The phenotype and multipotential differentiation of MSC cells used in the study has been verified (Additional file 1: Figure S1–2). Then BM-MSCs were treated with recombinant human TGF-β1 (10ng/ml, R&D Systems, Minneapolis, MN, USA) to trigger CAF differentiation. Conditioned medium from the CAFs or MSCs was obtained from cells cultured in regular medium with 1 % FBS. The leukemia cell lines THP-1 and K562 (American Type Culture Collection, Manassas, VA, USA) were cultured in 1640 or low-glucose DMEM supplemented with 10 % fetal bovine serum (FBS) and penicillin-streptomycin (Gibco, Grand Island, NY, USA) at 37 °C in 5 % CO_2_. Additionally, where appropriate, the leukemia cells were pre-incubated with the TGF-β1 inhibitor SB431542 (Sigma, St Louis, MO, USA) for 2 h at 37 °C in 5 % CO2.

### Transwell co-culture of CAFs with leukemia cells

CAF cells (2 × 10^4^ initial cell count) were cultured for 24 h in the upper side of a transwell chamber partitioned by a polycarbonate membrane (8.0um pore size, Corning Incorporated, Costar). And 2 × 10^5^ THP-1and K562 cells were co-cultured in the lower part in 1640 or DMEM medium with 10 % FBS at 37 °C, 5 % CO_2_ for 48 h.

### Evaluation of leukemic cell viability

THP-1 and K562 cells from suspension cultured, direct co-cultured or transwell co-cultured groups were treated with Ara-C (10uM, TCI Company, Tokyo chemical industry, Tokyo, Japan) for 48 h. Directly co-cultured THP-1/K562 cells were separated from the MSCs or CAFs monolayer by careful pipetting. After the leukaemic cells were collected, the monolayer was observed under the microscope (100×) to confirm that the monolayer was not damaged and that fewer than 10 leukaemic cells/vision field remained attached. Cell viability was evaluated by trypan blue exclusion in triplicate samples and quantified by a Cellometer Mini (Nexcelom, Lawrence, MA, USA).

### Immunohistochemistry and the reticulin fiber density (RFD) assay

The BM tissue samples were used for immunohistochemistry and Gomori’s stain. The rabbit anti-FSP1/S100A4 monoclonal antibody (Millipore, Billerica, MA, USA), rabbit anti-FAP polyclonal antibody (Abnova, Taipei, Taiwan), goat anti-α-SMA polyclonal antibody (Abcam, Cambridge, MA, USA), rabbit anti-GDF15 monoclonal antibody (Cell Signaling, Danvers, MA, USA), mouse anti-Vimentin monoclonal antibody (Santa Cruz, Dallas, CA, USA), and mouse anti-CD68 monoclonal antibody (Abcam) incubations were performed according to the manufacturer’s instructions. Images of the slides stained for these markers were scanned at 40× magnification using the optical microscope (Olympus Co., Tokyo, Japan). The positive rate of each marker was quantified by counting the ratio of the positive cells in all nuclear cells. The reticulin fiber density (RFD) of the samples was assessed according to Norén-Nyström et al [18]. All readings and estimations were performed in a blinded manner.

### Flow cytometry

The Alexa Fluorescence488 (AF488)-conjugated monoclonal antibodies specific for α-SMA (R&D Systems, Minneapolis, MN, USA), a rabbit anti-FAP polyclonal antibody (Abcam, Cambridge, MA, USA) and a FITC-conjugated anti-rabbit polyclonal antibody were used (eBioscience, San Diego, CA, USA). The appropriate isotype-matched antibodies were used as controls (R&D Systems). For the apoptosis assay, THP-1 and K562 cells (5 × 10^5^) were stained with Annexin-V and propidium iodide (PI) for apoptosis detection (BD, Franklin Lakes, New Jersey, USA) according to the recommended protocol on a Becton Dickinson Flow Cytometer. For cell cycle analyses, THP-1 cells were treated with RNase A and PI (Sigma, St Louis, MO, USA). The measurements were made using a flow cytometer (Beckman, Urbana, IL, USA).

### Quantitative reverse transcription PCR (qRT-PCR)

Total RNA was extracted using Trizol (Invitrogen, Paisley, UK), and the RNA was converted into cDNA using the Primer Script TM RT reagent Kit (Takara Bio Inc, Otsu, Shiga, Japan). All real-time PCR reactions were performed using an ABI 7500 real-time PCR system (Biosystems, Foster City, CA, USA) and the SYBR Premix Ex Taq reagent kit (Takara Bio Inc).

### Western blotting and ELISA

MSC or CAF cells were harvested in a radioimmunoprecipitation assay buffer for western blotting analysis (Beyotime, Haimen, Jiangsu, China). Mouse anti-collagen I monoclonal antibody (Abcam, Cambridge, MA, USA), rabbit anti-collagen III polyclonal antibody (Abcam), rabbit anti-FAP polyclonal antibody (Abnova, Taipei, Taiwan), rabbit anti-α-SMA monoclonal antibody (Epitomics, Burlingame, CA,USA), rabbit anti-GDF15 polyclonal antibody (Bioworld, Louis Park, MN,USA) and mouse anti-β-actin monoclonal monoclonal antibody (Cell Signaling, Danvers, MA, USA) were used. Antigen detection was performed using a goat anti-rabbit or an anti-mouse IgG secondary antibody (Cell Signaling) conjugated to HRP and an ECL western blotting substrate (Pierce, Waltham, MA, USA). The BM aspirates were collected from newly diagnosed AML patients which were divided into CR group (*n* = 9) and refractory group (*n* = 8) and normal controls (*n* = 11). The ELISA analysis for GDF15 was performed according to the manufacturer’s instructions (R&D systems).

### RNA interference

GDF15 siRNA and the control siRNA products were purchased from Gene Pharma Company (Suzhou, Jiangsu, China). Transfection of siRNA was performed using Lipofectamine2000 (Panc05.04) or Lipofectamine RNAiMax (Invitrogen, Paisley, UK) following the manufacturer’s protocol. The validation of target gene knockdown was performed using qRT-PCR using the delta-delta CT method.

### Statistical analyses

A Mann-Whitney U test was applied to compare the RFD of the AML patients and the normal controls. The statistical analyses were performed using the two-sample two-tailed Student’s t-test. The survival curves were constructed using the Kaplan–Meier method, and the log-rank test was used to test the difference in RFS and OS between cases with different RFD values. RFS was defined as the time between diagnosis and the onset of the first relapse, and OS was defined as the time between diagnosis and the occurrence of death or when lost to follow-up. Independent prognostic parameters for OS and RFS were identified by univariable Cox regression analyses and a multivariable Cox regression model using backward elimination with an exclusion significance level of 5 %. The data are described as the mean ± standard deviation (SD), and p values ≤0.05 were considered significant. All statistical analyses were conducted by using the SPSS 18.0 software program (SPSS, Chicago, IL, USA).

## Results

### Characteristics of the patients

The median ages of patients in the refractory group (*n* = 25) and CR group (*n* = 38) were 53 years (range: 23-82years) and 60 years (range: 18–80years) respectively. The ratios of male/female in these two groups were 18/7 and 27/11 respectively (Table 1). There were no statistical differences in age, sex and FAB subtypes between the two groups.

### RFD is higher in AML and correlates with the chemotherapy outcome

**Fig. 1:**
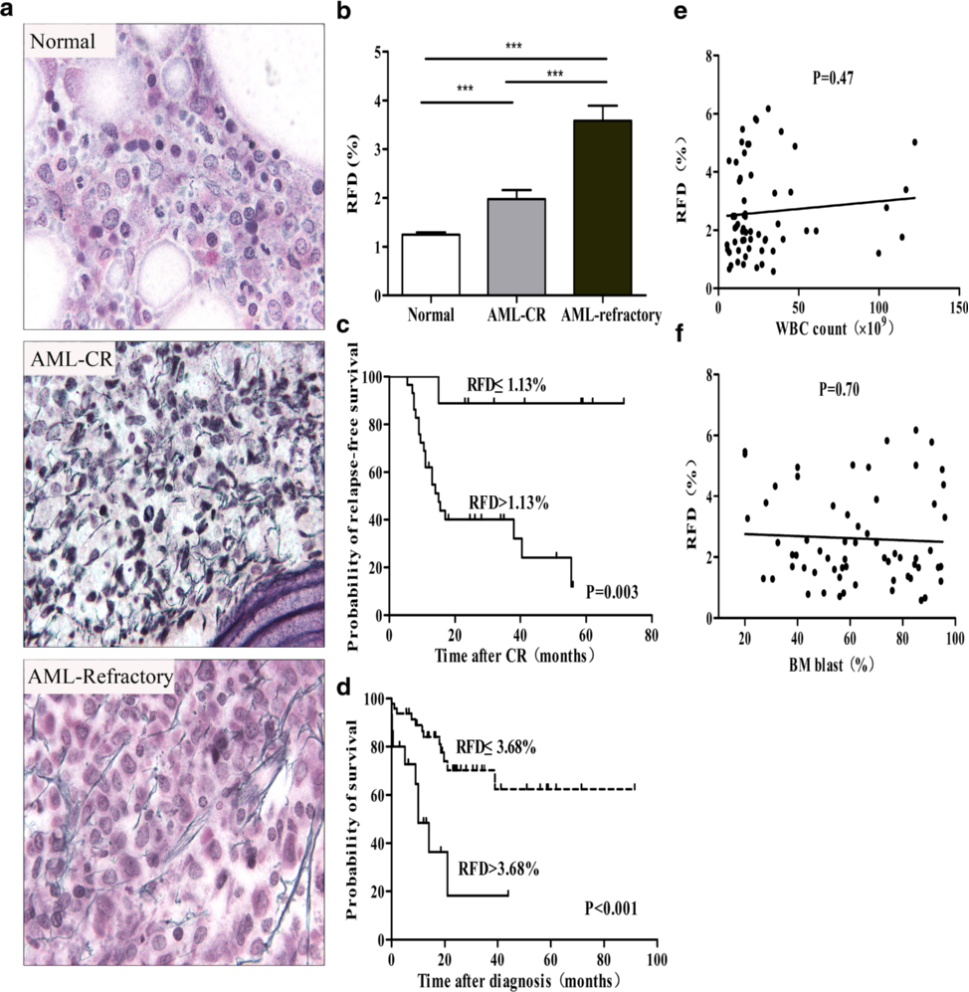
The RFD score is higher in AML and is related to outcome. **a** Examples of the Gomori staining of reticulin fibers in the BM sections from normal controls and AML patients, which were further divided into CR and refractory groups. Both images are at a magnification of 1000×. **b** The RFD for the AML-CR (*n* = 38) and AML-refractory (*n* = 25) patients compared to the normal controls (*n* = 59), *** *p* < 0.001 (**c**) RFS of AML-CR patients subdivided into high (>1.13 %) and low (≤1.13 %) RFD groups, *P* = 0.003. **d** OS of all the AML patients subdivided into high (>3.68 %) and low (≤3.68%) RFD groups, *P* < 0.001. **e**-**f** Scatter plot: RFD vs. blasts in the peripheral blood and the blasts in the BM of primary AML patients using linear regression analysis (*P* = 0.47 and 0.70 respectively)

Some data have indicated that increased fibrosis in the BM is a physical barrier against the effective delivery of therapeutic agents in childhood acute lymphoblastic leukemia (ALL) [19]. In our study, Gomori staining showed that abnormally proliferated reticular fiber, shown as black silk filaments, was distributed more densely in refractory AML patients (Fig. 1a). The RFD score in the refractory patients was higher when compared to the CR group, and both of them were significantly higher than the control (Fig. 1b). Furthermore, the Cox’s regression model showed that only the factor of RFD was associated with higher risk of relapse and death (*P* = 0.029 and 0.000, respectively) (Table 2). This was further illustrated in a Kaplan-Meier survival analysis. The cutoff used for low and high RFD was decided by exploring RFD data in Cox’s analyses dividing the material by the median and quartiles. In the AML-CR group, there was a poorer prognosis in RFS for cases with RFD > 1.13 % (Fig. 1c). And in all the AML patients, we also concluded that patients with RFD score of more than 3.68 % revealed a shortened overall survival (Fig. 1d). However, there was no correlation between the RFD score and blasts either in the peripheral blood or in the BM (Figs. 1e-f), suggesting that the fibers neither anchor the leukemia cells in the BM nor promote blast proliferation. Therefore, we hypothesized that the fibers may not directly affect AML patient prognosis, although the RFD score indicated a poor outcome in AML patients. Based on this information, we focused on the reticulin fiber-derived cells.

**Table 2:** Cox regression for overall survival (OS) and relapse-free survival (RFS)

	RFS				OS			
Variable	*n*	HR	95% CI	*P*	*n*	HR	95% CI	*P*
PWBC (10^9^/L)	38	1.009	0.988-1.030	0.420	63	1.003	0.988-1.019	0.674
Age (years)	38	0.950	0.974-1.025	0.950	63	1.002	0.976-1.030	0.862
BMP (%)	38	0.987	0.965-1.010	0.275	63	0.990	0.971-1.010	0.330
RFD (%)	38	1.400	1.035-1.892	0.029	63	1.755	1.331-2.312	0.000

*Abbreviations*: *PWBC* peripheral white blood cell, *BMP* Bone marrow precursors, *RFD* reticulin fiber density, *HR* hazard ratio, *CI* confidence interval

### The reticulin fibers were mainly derived from the CAFs in AML

**Fig. 2:**
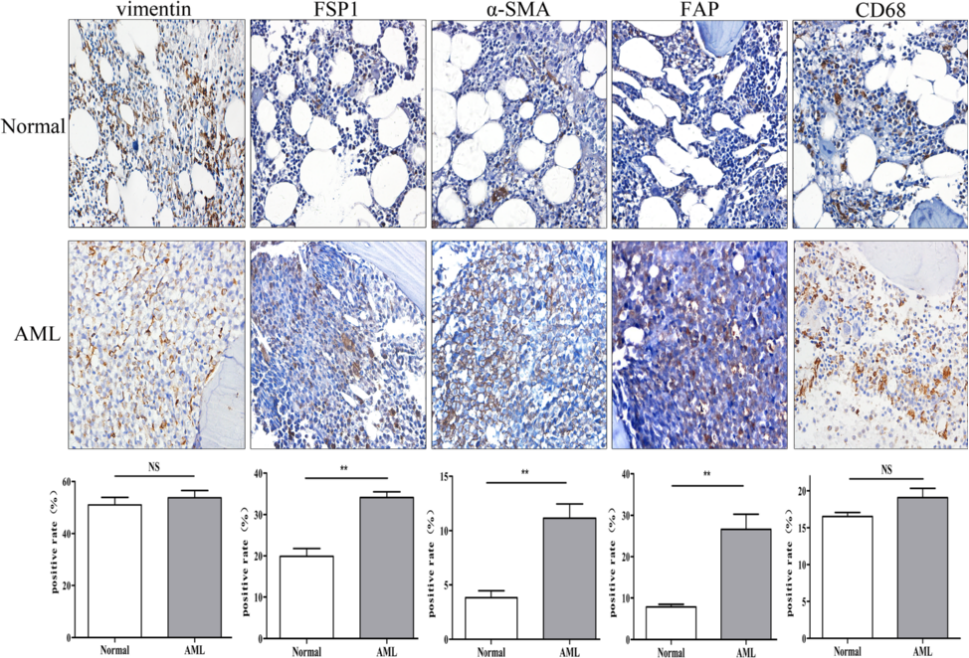
The reticulin fibers were mainly derived from CAFs in AML. Microphotographs displaying IHC staining for Vimentin, FSP1, α-SMA, FAP and CD68 in the BM sections of the normal controls and primary AML patients. Both images are at a magnification of 400×. The horizontal bar below the images indicates the percentage of positive cells that expressed Vimentin, FSP1, α-SMA, FAP and CD68 in the BM sections between these two groups (*n* = 3),** *p* < 0.01

The fibers in the BM were derived mainly from mesenchymal cells which were a heterogeneous population mainly comprised of adipocytes, macrophages and fibroblasts [20–22]. As we all know that adipocytes are seldom found in the BM of primary AML, therefore, adipocytes were not assessed in the survey. The mesenchymal markers Vimentin, FSP1, α-SMA and FAP were immunoassayed to identify fibroblasts, and macrophage cells were identified by CD68. As illustrated in Fig. 2, Vimentin, as a common marker of mesenchymal cells, showed no difference in the BM from AML patients and controls, indicating that the increased fibers in AML may originate from some special subpopulations of mesenchymal cells. However, there represented no difference in the positive rate of CD68 in the BM between these two subgroups, indicating that macrophages did not contribute to fibrosis in AML. Among the other mesenchymal markers, there was a significant increase in the cells positive for FSP1, α-SMA or FAP in the AML samples compared to the controls, and these are common markers of CAFs [23]. We thus hypothesis that the amount of CAFs strongly contributed to the increased fibers in AML, and we further to explore whether CAFs could play particular functions in AML progression.

### CAFs protect leukemia cells from chemotherapy

**Fig. 3:**
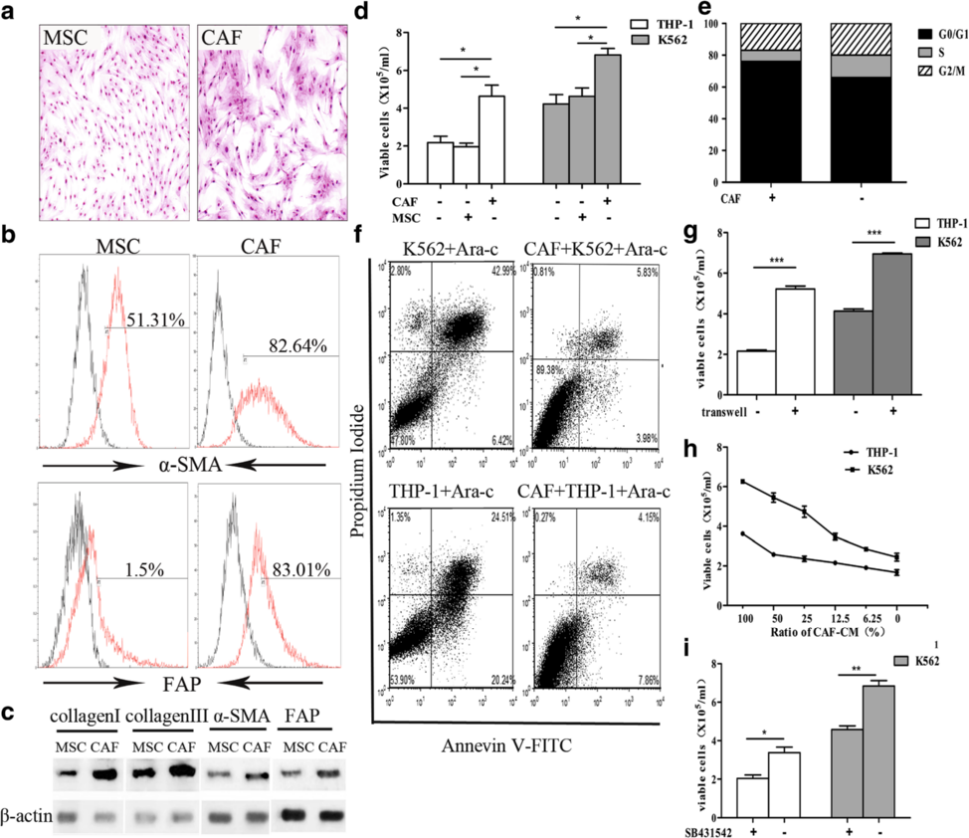
CAFs protect leukemia cells from chemotherapy **a** Morphological observations of the MSCs and the CAFs. Both images are at a magnification of 100×. **b** Flow cytometry analysis showed expression levels of a-SMA and FAP in the MSCs and the CAFs. **c** Western blotting for collagen I, collagen III, FSP1, α-SMA and FAP in the MSC and the CAF cells. **d** Bar plots illustrating the viability of THP-1 and K562 cells which were cultured in medium alone or direct co-cultured with MSCs or CAFs under treatment of Ara-C (10uM) for 48 h. **e** Cell cycle of the residual THP-1 cells which were treated with Ara-C (10uM) for 48 h when co-cultured with CAFs or not were analysized by propidium iodide staining and flow cytometry. **f** Apoptosis of THP-1 and K562 cells which were co-cultured with CAFs (right) or not (left) were evaluated by AnnexinV/PI staining and flow-cytometry. Data were presented for one of the triplicate experiments. **g** Bar plots showed the viability of THP-1 and K562 which were cultured in medium alone or co-cultured with CAFs in a transwell system under treatment of Ara-C (10uM) for 48 h. **h** The THP-1 and K562 cells were cultured in medium with different ratios of CAF conditioned medium (CAF-CM) under treatment with Ara-C (10uM) for 48h. **i**: The viable cells of THP-1 (white bar) and K562 (gray bar) which were co-cultured with a CAF layer under Ara-C treatment for 48h after pretreated with SB431542 or not were evaluated by trypan blue exclusion assay Data were presented as mean ± SD of triplicate experiments.* *p* < 0.05,** *p* < 0.01, ***p < 0.001

We focused on the protective effects of the CAFs in the leukemia cells from chemotherapy. As illustrated in Fig. 3a-c, under TGF-β1 treatment, the stroma cells exhibited a larger volume and an increase in cytoplasmic content and uniformly expressed α-SMA and FAP on the cell membrane as assessed by flow cytometry. These cells also had the capacity to synthesize higher levels of collagen I, collagen III, α-SMA and FAP protein as measured by western blotting, which further confirmed that MSCs could be transformed into CAF cells by TGF-β1. Co-cultured experiments showed that the mean survival of the CAF co-cultured leukemia cells was significantly increased compared with the cells co-cultured with the MSC or cultured alone (Fig. 3d). Leukemia cells co-cultured with the CAFs exhibited less apoptosis as determined by AnnexinV/PI staining analysis (Fig. 3f), suggesting that CAF reduce the sensitivity of the leukemia cells to chemotherapeutic agents. Moreover, the cell cycle status of the residual leukemia cells revealed an increased number of cells which arrested in the G0/G1 phase, indicating that the CAFs protected the leukemic cells by maintaining them in a quiescent phase thus decreasing their chemotherapy sensitivity (Fig. 3e). To verify the effect of soluble factors secreted by CAFs on the chemotherapy sensitivity of the leukemia cells, transwell experiment was carried out to prevent direct contact but permit exchange of soluble diffusible factors. As shown in Fig. 3g, the viable cell numbers in the transwell system were significantly increased when compared to the suspension cultured subgroup (*p* < 0.001). Furthermore, when the leukemia cells cultured in the CAF-CM as described in the methods above, the viability of the leukemia cells was decreased when the ratio of the CAF-CM was reduced (Fig. 3h). SB431542, which is considered to be a TGF-β receptor (ALK4/5/7) inhibitor, could partly abolished the protective effects of the CAFs for the tumor cells from chemotherapy (Fig. 3i), indicating that the chemo-protection for AML cells from CAF was mediated by some soluble substances, most likely TGF-β superfamily.

### GDF15 contributes to the CAF-mediated chemo-protection of AML cells

**Fig. 4:**
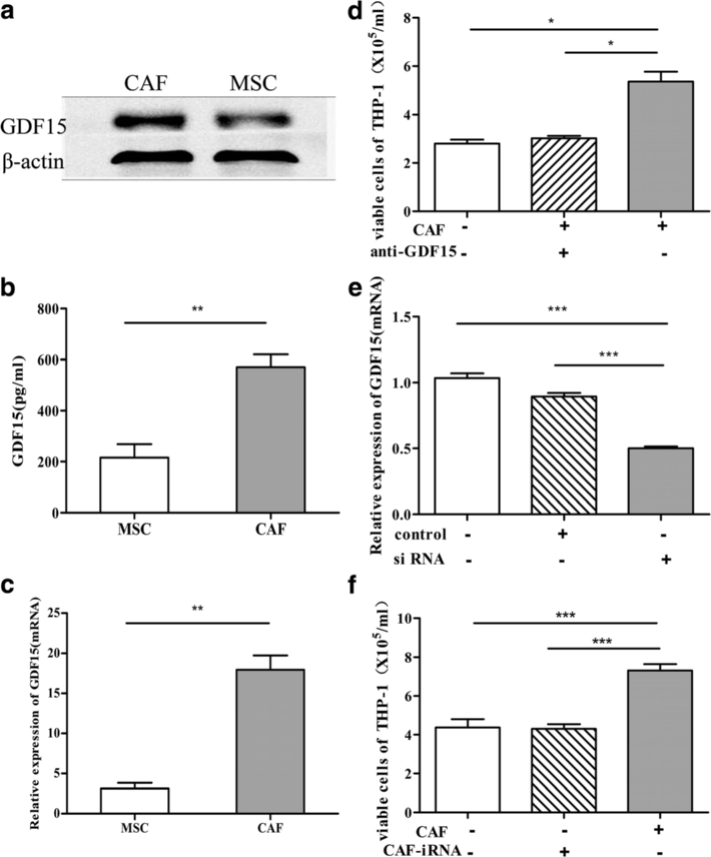
GDF15 contributed to the CAF-mediated chemo-protection of the AML cells. (**a**-**c**) Western blot, ELISA and Quantitative RT-PCR analyses represented GDF15 expression in the MSC and the CAF cells. **d** The bar plots showed the relative viability of the THP-1 cells cultured in medium alone or co-cultured with CAFs added with or without an anti-GDF15 antibody under the treatment of Ara-c (10uM) for 48 h. **e** Quantitative RT-PCR showing GDF15 expression in the CAFs after nucleofection of a GDF15-specific siRNA or a non-targeting control siRNA for 48 h. **f** The number of viable THP-1 cells cultured in medium alone or direct co-cultured with the layer of CAFs after nucleofection of GDF15-siRNA or not under the treatment of Ara-C (10uM) for 48 h. The data are the result of three independent experiments and are presented as means ± SD,* *p* < 0.05, ** *p* < 0.01, *** *p* < 0.001

Based on our previous results showing that GDF15 was markedly up regulated in the residual leukemic cells after chemotherapy [10] and reports that GDF15 confers drug resistance to other cancer cells [24–27], we questioned whether CAFs in the BM could also play an important role by secreting GDF15. As shown in Fig. 4a-c, the GDF15 in the cell lysates of the CAFs was significantly increased when compared to MSCs and was further abundantly released into the culture supernatant, confirming that the CAFs secreted enough GDF15. To further investigate the functional roles of GDF15, the neutralizing anti-GDF15 antibody was added to the co-cultured system. As shown in Fig. 4c, under the treatment of Ara-c and the neutralizing anti-GDF15 antibody, the survival of THP-1 was decreased. In addition, we knocked down GDF15 in the CAFs with a small interfering RNA targeting GDF15 (siGDF15) and then validated by RT-PCR analysis (Fig. 4d). As shown in Fig. 4e, these CAF cells nearly lost their potential to protect THP-1 cells from chemotherapy. Our results demonstrating that GDF15 substantially contributes to the CAF-mediated chemo-protection of AML cells.

### GDF15 in the BM of AML patients

**Fig. 5:**
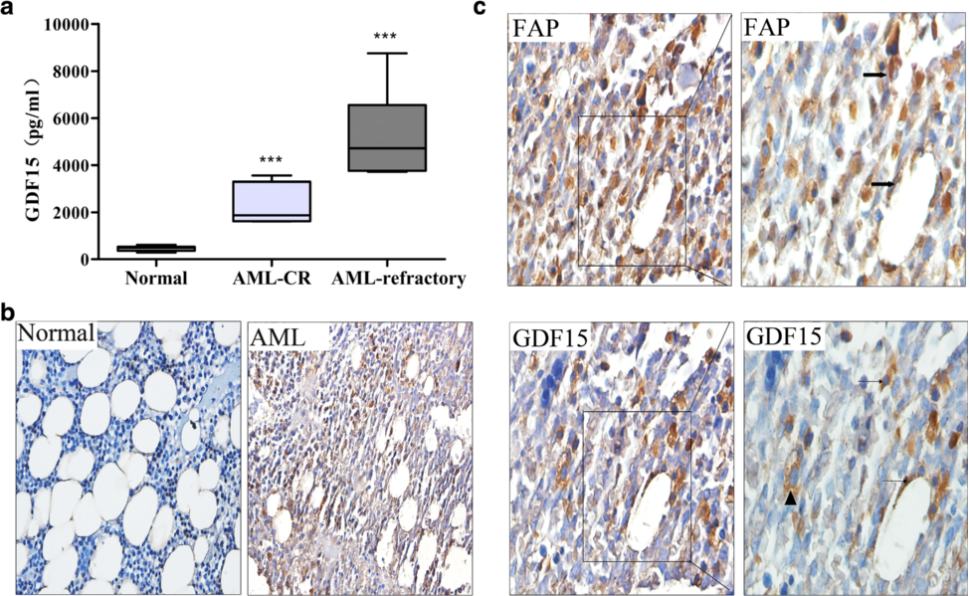
GDF15 expressed in the BM of AML in vivo. **a** ELISA analysis of GDF15 levels in the BM aspirates of the controls (*n* = 11) and treatment-naïve AML-CR (*n* = 9) and AML-refractory patients (*n* = 8) *** *p* < 0.001vs the control group. **b** Examples of immunohistochemistry for GDF15 in the BM sections of the normal control and the primary AML patient. Both images are at a magnification of 400×. **c** Immunohistochemistry identifies cells that express FAP (arrows) and GDF15 (arrowheads) in the AML patients. GDF15 could also be detected in the leukemia cells (triangle). The images on the left are at a magnification of 400× and on the right are at a magnification of 1000×

To further confirm the pathophysiological contribution of GDF15 in CAFs, the amount of GDF15 in the BM aspirates was compared between the normal controls and the primary AML patients. To avoid the confounding effects of therapy-induced elevations of GDF15, the BM aspirates were derived from treatment-naïve patients. As shown in Fig. 5a, GDF15 in the BM aspirates from AML-refractory group was higher than the AML-CR group and both of them were significantly higher than the control group. To further investigate the distribution of GDF15 in the BM, immunohistochemistry analyses were performed in the BM biopsies. As illustrated in Fig. 5b, GDF15 was constitutively expressed in the cytoplasm of spindle cells, which were clearly observed in the BM biopsies from the AML patients, whereas these cells were scarcely in the controls. In addition, immunoassays on the serial sections showed that these GDF15^+^ cells were mainly co-localized with FAP, which was identified as the typical immunophenotype of the CAFs (Figs. 5c). These results confirmed the possibility that GDF15 contributes to the CAF-mediated chemotherapy protection for leukemia cells.

## Discussion

As it is known that the normal tumor microenvironment becomes ‘corrupted’ during tumor development, which is reflected by appearance of a large and heterogeneous category of cancer-associated fibroblasts (CAFs) [28]. CAF cells are considered active modulators of the tumor microenvironment among many solid tumors [29–31]. However, few studies have directly addressed the role of the CAFs in the BM of leukemia. In the present study, we demonstrate that functional CAFs are widespread in the BM of AML patients and could serve as a critical chemo-protective element for AML cells by producing an abundance of GDF15.

As we all know, AML cells interact both anatomically and functionally with the stroma within the BM microenvironment. These interactions have a critical role in the development, progression and relapse of AML. A recent study has suggested discoidin domain receptor 1 (DDR1), a class of collagen-activated receptor tyrosine kinase (RTK) was highly upregulated on bone marrow (BM)-derived CD33+ leukemic blasts of acute myeloid leukemia (AML) patients, suggesting that the remodeled collagen IV in BM microenvironment could modulate the migration and adhesion of myeloid leukemia cells during leukemogenesis [32]. In this study, the RFD and the immunohistochemistry assay were used to detect the transformation of the stroma niche in BM during acute leukemogenesis. It has been reported that reticulin in BM is mainly composed of individual fibrils or small bunches of fibrils of type III collagen surrounding a core of type I collagen fibrils [20]. Our experiments also confirmed the presence of the amounts of collagen I and collagen III in the marrow of AML (Additional file 1: Figures S3). This result is in agreement with those from previous studies, which have revealed that CAFs might produce and secrete various extracellular matrix proteins (i.e., collagen I, III, IV) in solid tumors [33]. Data has been proposed that CAFs are responsible for collagen synthesis in the stroma of human hepatocellular carcinoma [34]. But reports about the origin of the fiber is still rare, to our knowledge, Jean Y P et al. firstly developed the quantitative dissection of stromal cell-extracellular matrix interactions in living tissue by using multiphoton laser scanning microscopy and second harmonic generation (SHG) of fibrillar collagen. They showed that the CAF is necessary for fiber remodeling [35]. Based on the support from the literatures, we considered that the increased fiber stroma in BM might imply the increased of CAFs during leukemogenesis. Positive staining for the CAF-specific markers α-SMA, FAP and FSP1 could also displayed that amount of the CAFs contributed to the abundant fibers in the BM. In addition, there existed sufficient TGF-β1 in BM microenvironment of leukemia to stimulate the formation of CAF niche (Additional file 1: Figure S4).

Resistance to anti-cancer therapies which is the major obstacle to a better prognosis of patients obtained increasingly attention in the modern biomedical research [36]. Acting on cancer cells, CAFs have previously been reported to be involved in resistance to chemotherapy in many solid tumors. Our co-cultured experiments showed that these functional fibroblasts could also exhibit a chemo-protective effect on the leukemia cells. It is worth noting that our results did no find the similar protective effects of the stroma cells which were derived from the healthy donors and pretreated with TGF-β1(10ng/ml) for 14 d (Additional file 1: Figure S5). We suspected this may be due to the differences in BM-MSC themselves between the AML patients and the healthy ones. The mechanisms involved in the anti-chemotherapy effects of CAFs on AML cells are not clear. In this report, we detected that either knockdown of GDF15 in the CAFs or interference by neutralizing anti-GDF15 in the co-culture system decreased the mortality of the THP-1 cells, confirming that GDF15 has an essential function in the CAF-mediated chemotherapeutic resistance of the leukemic cells. When compared with MSC and the THP-1 cells, we detected an increased GDF15 concentration in the conditioned medium of the CAFs (Additional file 1: Figure S6). Therefore, we assumed that CAFs were likely to be the main contributor to the high concentrations of soluble GDF15, which was different form our previous publication that GDF15 derived from leukemia cells conferred chemoresistance in acute lymphoblastic leukemia, we suspect there existed originally differences in the lymphoid and myeloid leukemia cells. As we all know that GDF15, as an important component of the TGF-β superfamily, is notably increased in patients with prostate, colorectal, or pancreatic cancers and has been described as an important biomarker of poor clinical outcome [37–39]. However, the GDF15 concentrations in the BM of AML patients are not well defined. In our experiments, the direct evidence for the CAFs contributing to high GDF15 expression is supported by the BM biopsies, which indicated that this factor was mainly expressed by the CAFs and colocalized with FAP by immunohistochemistry. GDF15 is present both on the membrane and in the cytoplasm of leukemic cells (Fig. 5c), suggesting that leukemia cells have an autocrine function, which is consistent with the results of ELISA analysis of the leukemia cell lines in vitro. Both the CAFs and the leukemia cells secreted GDF15, suggesting that GDF15 plays a critical role in the local cross-regulation between the leukemia cells and the CAFs within the BM in primary AML patients. However, the detection of CAF cells on BM biopsies were not routinely performed in AML patients, so if there resist a correlation between CAF density and AML survival still remains unclear. Based on this, it is necessary for us to construct the mouse transplantation models of AML to explore the correlation between CAF density and further to explore the potential mechanisms in vivo.

## Conclusions

This study highlights the importance of the fiber stroma in leukemogenesis. The abundant reticulin fiber predicts a poor outcome of AML. Based on the experiments, we suggest that a portion of the CAF cells exist in the BM microenvironment of AML. And these functional cells affected the chemosensitivity of the leukemia cells by producing GDF15. Disrupting GDF15 via treatment with an anti-GDF15 antibody or si-GDF15 in the CAFs abrogated the protection of the leukemic cells by CAFs. To our knowledge, this is the first report of the molecular mechanisms of CAFs in AML. We suspect that there might maintain a chemo-protective niche in a pool of AML cells causes the relapse, which may need for further animal experiments to verification. In this regard, future studies from our laboratory will assess GDF15 as a novel target for therapeutic strategies in AML in vivo by using a mouse transplantation model of AML.

## Additional file

Additional file 1: Figure S1. Phenotype characterization of BM-MSCs. Flow Cytometry analysis showed that almost all the cultured MSC expressed CD90, CD105 and CD73, while a small portion of MSC expressed CD14, CD34 and CD45. **Figure S2.** Differentiation potential of BM-MSCs. (a) MSC differentiation to adipocytes was shown by Oil-O-Red staining. (B) And osteoblast differentiation was detected by Alizarin Red staining. The images are at a magnification of 200×. **Figure S3.** Immunohistochemistry to detect the expression of collagen I and collagen III in the BM of the normal control and the AML patient. Both images are at a magnification of 400×. **Figure S4.** The levels of TGF-β1 in BM plasma between AML patients and the normal controls were analyzed by the Quantikine ELISA kit ((R&D Systems, USA), according to the manufacturers instruction. The absorbance at 450 nm was detected by the microplate reader. Concentrations were calculated from the constructed linear curve. Data are presented as the mean ± standard deviation; *n* = 5 per group. ******* *p* < 0.01. **Figure S5.** Bar plots illustrating the viability of leukemia cells (THP-1 and K562) under treatment of Ara-C (10uM) for 48 h. THP-1/K562 cells were cultured in medium alone or co-cultured with the stroma cells derived from BM-MSC of healthy donors and pretreated with TGF-β1(10ng/ml) or not. The error bars represent the standard error of the mean of three replicates, ns, *p* > 0.05. **Figure S6.** The horizontal bar showed Elisa analysis of GDF15 in the supernatants of the MSC, THP and CAF cells. The data represent the mean ± SD of triplicate experiments, ****** *p* < 0.01. (DOC 6094 kb)
