# Supplementary material for: Growth differentiation factor 15 contributes to cancer-associated fibroblasts-mediated chemo-protection of AML cells
Source: J Exp Clin Cancer Res. 2016 Sep 19;35:147. doi: 10.1186/s13046-016-0405-0 (PMC5029001; doi:10.1186/s13046-016-0405-0)

**Additional file1:**

**Figure S1:** Phenotype characterization of BM-MSCs. Flow Cytometry analysis showed that almost all the cultured MSC expressed CD90, CD105 and CD73, while a small portion of MSC expressed CD14, CD34 and CD45.

**Figure S2:** Differentiation potential of BM-MSCs. (a) MSC differentiation to adipocytes was shown by Oil-O-Red staining. (B) And osteoblast differentiation was detected by Alizarin Red staining. The images are at a magnification of 200x.

**Figure S3:** Immunohistochemistry to detect the expression of collagen I and collagen III in the BM of the normal control and the AML patient. Both images are at a magnification of 400x.

**Figure S4:** The levels of TGF-β1 in BM plasma between AML patients and the normal controls were analyzed by the Quantikine ELISA kit ((R&D Systems, USA), according to the manufacturers instruction. The absorbance at 450 nm was detected by the microplate reader. Concentrations were calculated from the constructed linear curve. Data are presented as the mean ± standard deviation; n=5 per group. ******* p<0.01.

**Figure S5:** Bar plots illustrating the viability of leukemia cells (THP-1 and K562) under treatment of Ara-C (10uM) for 48 h. THP-1/K562 cells were cultured in medium alone or co-cultured with the stroma cells derived from BM-MSC of healthy donors and pretreated with TGF-β1(10ng/ml) or not. The error bars represent the standard error of the mean of three replicates, ns, p＞0.05.

**Figure S6:** The horizontal bar showed Elisa analysis of GDF15 in the supernatants of the MSC, THP and CAF cells. The data represent the mean ± SD of triplicate experiments, ****** p<0.01.


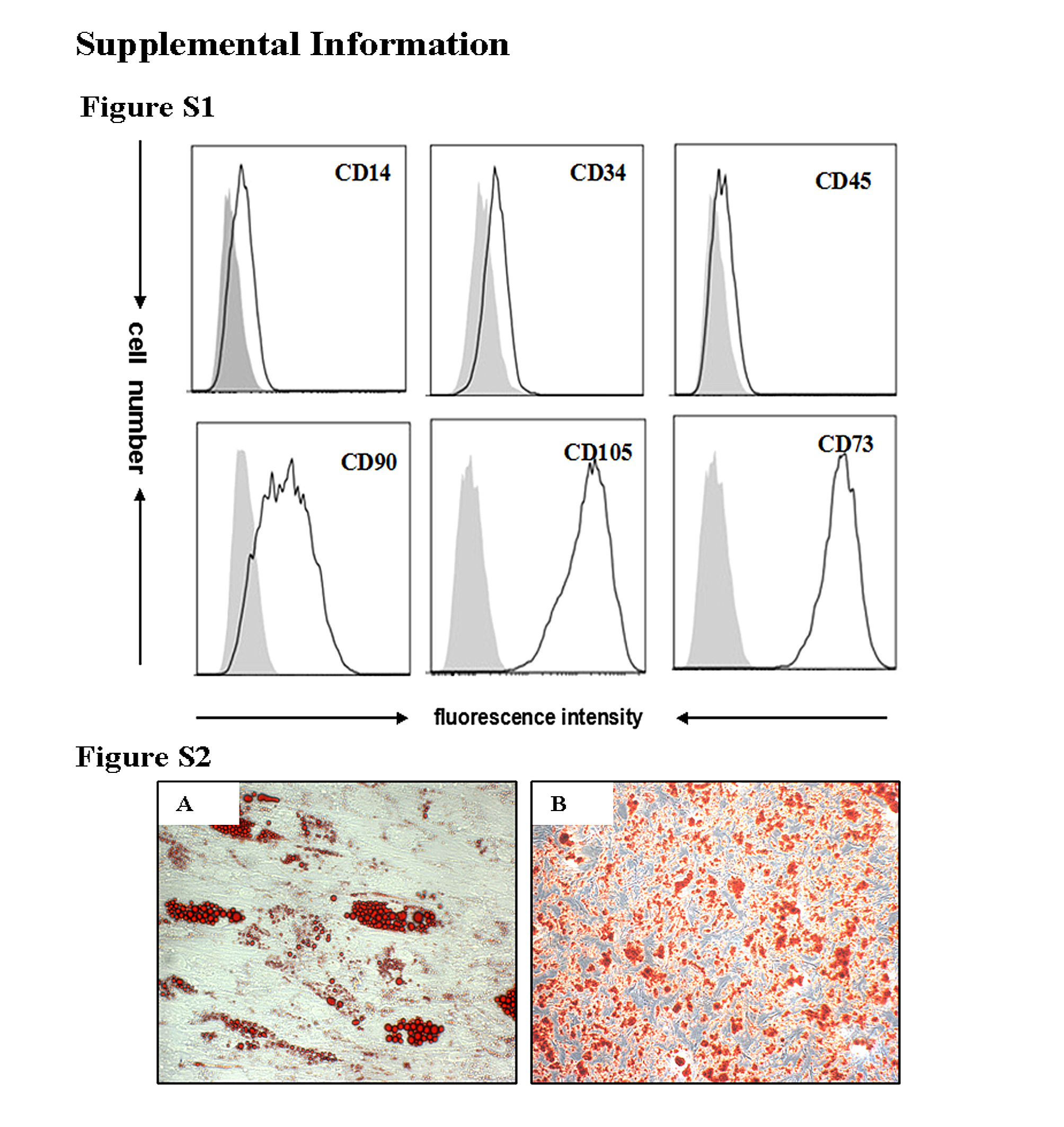


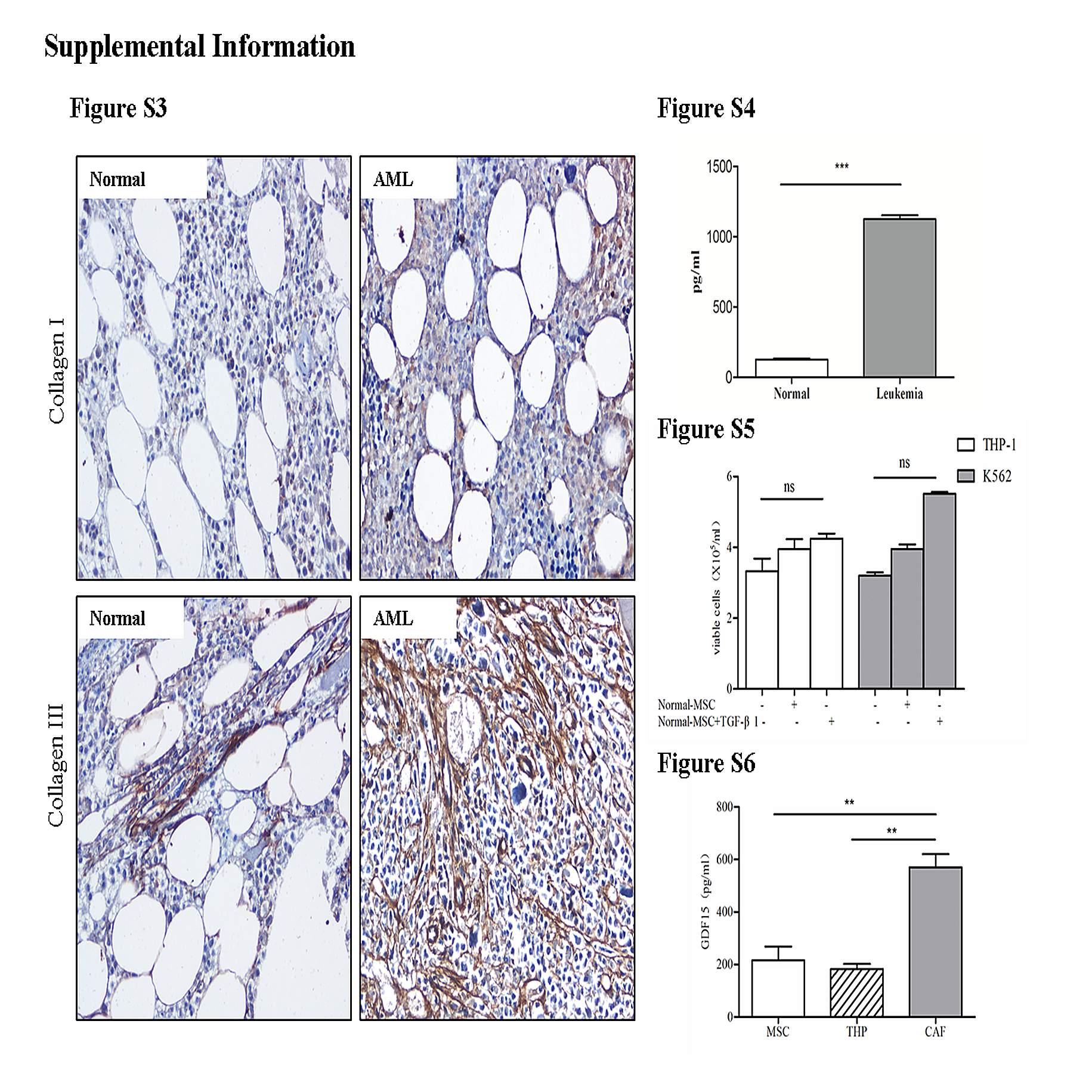

Supplement: Additional file 1: Figure S1. — Phenotype characterization of BM-MSCs. Flow Cytometry analysis showed that almost all the cultured MSC expressed CD90, CD105 and CD73, while a small portion of MSC expressed CD14, CD34 and CD45. Figure S2. Differentiation potential of BM-MSCs. (a) MSC differentiation to adipocytes was shown by Oil-O-Red staining. (B) And osteoblast differentiation was detected by Alizarin Red staining. The images are at a magnification of 200×. Figure S3. Immunohistochemistry to detect the expression of collagen I and collagen III in the BM of the normal control and the AML patient. Both images are at a magnification of 400×. Figure S4. The levels of TGF-β1 in BM plasma between AML patients and the normal controls were analyzed by the Quantikine ELISA kit ((R&D Systems, USA), according to the manufacturers instruction. The absorbance at 450 nm was detected by the microplate reader. Concentrations were calculated from the constructed linear curve. Data are presented as the mean ± standard deviation; n = 5 per group. *** p < 0.01. Figure S5. Bar plots illustrating the viability of leukemia cells (THP-1 and K562) under treatment of Ara-C (10uM) for 48 h. THP-1/K562 cells were cultured in medium alone or co-cultured with the stroma cells derived from BM-MSC of healthy donors and pretreated with TGF-β1(10ng/ml) or not. The error bars represent the standard error of the mean of three replicates, ns, p > 0.05. Figure S6. The horizontal bar showed Elisa analysis of GDF15 in the supernatants of the MSC, THP and CAF cells. The data represent the mean ± SD of triplicate experiments, ** p < 0.01. (DOC 6094 kb) [file 13046_2016_405_MOESM1_ESM.doc]
